# Reverse Genetic Analyses of Hydrophobins in *Sclerotinia sclerotiorum* Revealed Their Diverse Roles in Development, Environmental Survival, and Virulence

**DOI:** 10.3390/pathogens14111131

**Published:** 2025-11-06

**Authors:** Jinyi Tan, Zhengxi Gong, Xinyi Huang, Shawn D. Mansfield, Xin Li

**Affiliations:** 1 Michael Smith Laboratories, University of British Columbia, Vancouver, BC V6T 1Z4, Canada; 2 Department of Botany, University of British Columbia, Vancouver, BC V6T 1Z4, Canadashawn.mansfield@ubc.ca (S.D.M.); 3 Department of Wood Science, University of British Columbia, Vancouver, BC V6T 1Z4, Canada

**Keywords:** plant fungal pathogen, *Sclerotinia sclerotiorum*, hydrophobin, virulence, sclerotia, ascospore

## Abstract

*Sclerotinia sclerotiorum* is a notorious soilborne fungal pathogen that causes white mold in a wide range of host plants, leading to globally significant yield loss in many crops. Hydrophobins (HPs) are small, secreted proteins unique to filamentous fungi, with diverse roles in fungal biology. However, their functions in *S. sclerotiorum* remain poorly understood. Here, we systematically investigated the roles of three HP genes, *SsHP1*, *SsHP2*, and *SsHP3*, through reverse genetic analyses. By analyzing their deletion mutant phenotypes, we demonstrate that class I HP (*SsHP1*) is specifically required for proper sclerotia development, whereas class II HPs (*SsHP2* and *SsHP3*) are essential for compound appressoria functionality. All three HPs contribute to fungal surface hydrophobicity, cell wall integrity, and stress tolerance. Using mycelial fusion, we generated double mutants lacking both class II HPs, which exhibited more severe defects in appressoria development, virulence, cell wall integrity, and stress adaptation, indicating their partially redundant roles. SsHP2 is required for both host penetration and post-penetration virulence, whereas SsHP3 mainly affects host penetration, revealing their overlapping yet distinct contributions to pathogenic development. Although all HP mutants formed normal apothecia and asci, they released significantly fewer ascospores, suggesting that HPs are dispensable for sexual morphogenesis but crucial for the biophysical process of ascospore dispersal. Furthermore, carbohydrate analyses uncovered that these HPs affect cell wall composition, more broadly influencing stress adaptation and virulence. Taken together, our study reveals both conserved and divergent roles of HPs across fungi and highlight their multifaceted contributions to *S. sclerotiorum* biology, offering new perspectives for disease management.

## 1. Introduction

*Sclerotinia sclerotiorum* is a soilborne fungal pathogen, a representative species of the Sclerotineaceae family in Ascomycota [1,2]. It is one of the most devastating plant pathogens that causes substantial damage to major crops, including canola, vegetables, various beans, and sunflowers in Canada, the USA, and other countries [3,4]. Its widespread impact has resulted in substantial economic losses worldwide [1,3,5]. Traditional control methods are largely ineffective due to the pathogen’s broad host range and the general lack of resistance in crop cultivars. A key feature of *S. sclerotiorum* is the formation of sclerotia–a dormant structure encased in melanized surface layers [5,6]. Sclerotia serve two functions: acting as overwintering structures that allow the fungus to survive in unfavorable conditions and playing a crucial role in reproduction [1]. Sclerotia can germinate as mycelia, leading to direct plant infection. They can also produce apothecia that can release windborne ascospores to infect nearby hosts [1,7]. During infection, like other fungal pathogens, it can form complex appressoria structures for host cell wall penetration [1]. Despite its agricultural significance, the molecular mechanisms underlying *S. sclerotiorum*’s ability to sense extracellular stimuli, produce key developmental structures such as sclerotia, apothecia and appressoria, and interact with plant hosts remain largely unknown.

Hydrophobins (HPs) are small, secreted, hydrophobic proteins widely distributed in mycelial fungi [8,9]. They can self-assemble into robust, highly amphipathic polymeric monolayers [9,10,11]. HPs are characterized by the presence of eight conserved cysteine (Cys) residues, separated into two classes based on their amino acid sequences, hydropathy profiles, and solvent solubility. Class I HPs, generally longer, are commonly found in both ascomycetes and basidiomycetes, whereas class II HPs are more conserved and limited to ascomycete [12,13]. Class I HPs are able to assemble into a stable structure, known as rodlet layer, and they are only soluble in strong acids, such as trifluoroacetic acid (TFA) or formic acid (FA) [9]. In contrast, class II HPs cannot form the fibrillar rodlet morphology and can be dissolved in organic solvents and detergents such as 2% sodium dodecyl sulphate (SDS) or 60% ethanol [9,12].

HPs play essential roles in fungi by forming self-assembled amphipathic layers that lower surface tension, enabling aerial hyphae to breach the air-water interface [14,15,16]. They also render spores hydrophobic, promoting efficient dispersal, protecting against external environment, and facilitating surface attachment to surfaces—an important prerequisite for infection and germination [15,16,17,18]. Many fungi produce multiple HPs that function across different stages of the life cycle. For instance, the basidiomycete *Schizophyllum commune* encodes four HPs (SC1,SC3,SC4, and SC6) that are crucial for fruiting body development and for enabling hyphae to penetrate water or soil surfaces [11,19,20]. Among them, the well-characterized SC3 also contributes to the cell wall matrix, influencing its composition [21]. In ascomycete *Aspergillus nidulans*, six HPs (RodA and DewA–DewE) collectively mediate amphipathic layer formation and spore hydrophobicity, with RodA playing the dominant role [22]. Beyond development, HPs also participate in symbiotic interactions, including lichen symbiosis and the ectomycorrhizal association between *Tricholoma terreum* and its host trees, largely in the pine family, but also the spruces [23,24].

HPs have also been studied in several plant fungal pathogens, where they play essential roles in various aspects of fungal biology. In the model plant pathogen *Magnaporthe oryzae*, a class I HP (MPG1) and a class II HP (MHP1) have been identified. These proteins are involved in surface hydrophobicity, conidiation, appressorium development, as well as pathogenicity [25,26]. MPG1 is particularly important for host recognition, mediating morphogenetic signals that trigger appressorium differentiation [25,27]. Mutants with *MPG1* disruption fail to form the rodlet layer on conidial walls and exhibit a water wettable phenotype; whereas *MHP1* deletion mutants only display detergent wettable phenotype [25,26]. These finding suggested that MoMPG1 may specifically be responsible for rodlet layer formation in *M. oryzae*, while MoMHP1 may support hydrophobicity through a rodlet-independent mechanism. In fungal corn pathogen *Fusarium verticillioides*, five *HP* genes have been identified. Among them, *HYD1* and *HYD2* are essential for the structural integrity of the long aerial chains of microconidia, a distinctive feature of this pathogen. However, none of these five HPs are essential for corn seedling infection [28]. Similarly, in the wilt fungus *Verticillium dahlia*, the class II HP VDH1 is required for its fungal development, including microsclerotia development and spore viability, but is not necessary for the initiation of disease in tomatoes [29].

In the airborne plant pathogen *Botrytis cinerea*, three *HP* genes have been studied. Individually, these genes are not required for conidia and hyphae hydrophobicity and are not involved in its asexual development or pathogenicity [30]. However, double (*bhp1/bhp2*) and triple knock-out mutants exhibit abnormal apothecia development when used as maternal parents (sclerotia), suggesting the importance of HPs in fungal sexual development, particularly in maternal tissues [13].

*S. sclerotiorum* is closely related to *B. cinerea*, and are both in the Sclerotineaceae family. It also contains three *HP* genes in its genome [30]. However, the biological functions of these genes in *S. sclerotiorum* remain largely unknown. In this study, we classified and characterized *HP* genes in *S. sclerotiorum*, including *SsHP1* in class I, and *SsHP2* and *SsHP3* as class II members. Phenotypic analyses of HP mutants revealed that *SsHP1* plays a role in sclerotia development, while *SsHP2* and *SsHP3* are essential for proper compound appressoria function and virulence. Double mutant *Sshp2 Sshp3* exhibited pronounced defects in both appressoria development and virulence, indicating overlapping and critical roles in infection structure formation and host colonization. In addition, all HPs in *S. sclerotiorum* contribute to cell wall integrity, composition, and resistance to various abiotic stresses to different extents. Interestingly, these functions contrast with those reported in *B. cinerea*, where HPs are dispensable for virulence but required for sexual development, suggesting an evolutionary divergence of HP function even between closely related fungi. Taken together, our findings provide new insights into the multifaceted roles of HPs in fungal biology, highlighting class II HPs as potential targets for novel strategies in disease mitigation of *S. sclerotiorum*.

## 2. Materials and Methods

### 2.1. Fungal Strains and Culture Conditions

The wild-type (WT) strain *S. sclerotiorum* 1980 and all the knockout (KO) mutant strains generated in this background were cultured on potato dextrose agar (PDA, Shanghai Bio-way technology, Shanghai, China) at room temperature. Strains were maintained either on PDA slants at 4 °C or preserved as sclerotia. Selection and purification of KO mutants were conducted on PDA with hygromycin B (50 μg/mL, MilliporeSigma, Oakville, ON, Canada) to verify successful transformant isolation.

Carrot medium was prepared from sliced, autoclaved carrots and used to cultivate large sclerotia for apothecia production. Mycelia from PDA plates were inoculated onto the carrot medium and incubated at room temperature for 3–4 weeks until sclerotia matured.

### 2.2. Apothecium Induction

For apothecium formation, large sclerotia harvested from carrot medium were gently cleaned to remove adhering mycelia and carrot residues. After air-drying at room temperature, the sclerotia were immersed in 33% (*v*/*v*) bleach solution (Clorox Disinfecting Bleach, The Clorox Company, Oakland, CA, USA) for about 6 min for surface sterilization, followed by three rinses using sterile water to remove the remaining bleach. The sterilized sclerotia were then placed onto pre-autoclaved sands in containers covered with aluminum foil and incubated at 4 °C for approximately 4 weeks. After cold and dark treatment, the foil was replaced with alcohol-sterilized plastic wrap, and the containers were transferred to a plant growth chamber set at 16 °C with a 16 h light/8 h dark photoperiod. Apothecium development was typically observed at approximately 3–5 weeks.

### 2.3. Target Genes Knockout (KO)

The split-marker strategy was employed to construct *SsHP1*, *SsHP2*, and *SsHP3* gene replacement cassettes and generate KO mutants, following the procedure as previously described [31]. Briefly, the gene replacement fragment, containing the flanking sequences of the target gene surrounding the hygromycin-resistance gene (*HYG*), was introduced into WT *S. sclerotiorum* protoplasts. Transformants showing hygromycin B resistances were selected and were further purified through sequential tip mycelia transfers followed by protoplast purification. This process was repeated until PCR analysis with the 5F/6R primer pair confirmed the absence of WT band, verifying successful gene deletion. All the primers used in this study are listed in Supplemental Table S1.

The double mutant was generated using a previously described mycelial fusion method [32]. Mycelial disks of *Sshp2* and *Sshp3* single mutants were positioned in direct contact, face-to-face, and co-inoculated onto carrot medium to facilitate fusion and the development of large sclerotia. These large sclerotia were subsequently induced to produce apothecia, and single colonies derived from the resulting ascospores were screened on PDA containing hygromycin B. Putative double KO mutants were verified by PCR, where no WT-specific bands were detected using the 5F/6R primer pairs for both genes.

### 2.4. S. sclerotiorum Growth Rate and Colony Morphology

To evaluate growth rate and assess colony morphology, 5 mm mycelial agar disks were excised from the margins of 2-day-old WT and mutant colonies using a sterile pipette tip and placed at the center of fresh 85 mm PDA plates, which were then incubated at room temperature. For each genotype, three biological replicates were performed. Colony diameter was measured every 12 h until the plates were fully colonized. Colony morphology and sclerotia formation were documented by imaging the plates 7 days post-inoculation. The number of sclerotia was evaluated on two-week-old PDA plates, also with three biological replicates, and the sclerotia were subsequently collected for imaging. The same WT strain served as the control as the plates were grown at the same time. For clarity and consistency, identical WT images are presented across relevant figures for different mutants.

### 2.5. Plant Infection Assay

Fresh mycelial plugs (1 mm or 5 mm in diameter) taken from the colony margins of 2-day-old cultures were inoculated on detached leaves of approximately 4-week-old *A. thaliana* (Col-0) or *N. benthamiana*, with or without prior mechanical wounding. The inoculated leaves were placed on moistened paper towels inside trays covered with lids to ensure consistent humidity. The trays were then incubated in a growth chamber at 23 °C under a 16 h light/8 h dark condition. Lesion sizes were quantified using ImageJ software (1.53v).

### 2.6. Compound Appressoria Observation

Fresh mycelial plugs (5 mm in diameter) taken from the colony margins of 2-day-old cultures were placed onto glass slides and incubated on moistened paper towels in Petri dishes at room temperature for 2 days. Compound appressoria formation was examined using a ZEISS light microscope, and images were captured at 100× or 200× magnification. Scale bars are included in each image and described in the corresponding figure legend. The same WT strain served as the control as the experiments were carried out at the same time. For clarity and consistency, identical WT images are presented across relevant figures for different mutants.

### 2.7. Surface Hydrophobicity Assay

All *S. sclerotiorum* WT and HP mutant strains were cultured on PDA media for 10 days before experimentation. 10 μL of sterile distilled water, 0.2%SDS, and 2%SDS were placed on the surface of the plate culture. The results were observed and documented at different time points.

### 2.8. Oxalic Acid (OA) Accumulation Assay

Agar disks (5 mm in diameter) containing mycelia from WT and different mutant strains were excised from the edges of colonies using a sterile pipette tip and placed at the center of 85 mm Petri dishes containing PDA medium supplemented with bromophenol blue (which appears violet at pH > 4.6 and yellow when pH < 3.0). Plates were incubated at room temperature and color changes were documented. Each assay included three technical replicates.

### 2.9. Cell Wall Integrity and Stress Treatment Assay

Mycelial agar disks (5 mm in diameter) from the actively growing margins of WT and mutant colonies were obtained using a sterile pipette tip and placed at the center of 85 mm Petri dishes containing PDA medium supplemented with various stress agents: 5 mg/mL Congo Red (CR, Sigma-Aldrich, St. Louis, MO, USA), 0.01% Sodium Dodecyl Sulfate (SDS, BIO-RAD, Hercules, CA, USA), and osmotic stressors 1 M NaCl (BioShop, Burlington, ON, Canada)and 1 M Sorbitol (Thermo Fisher Scientific, Waltham, MA, USA). Plates were incubated at room temperature, and colony diameters were recorded after 48 h of growth under each stress condition. Colony morphology was also documented. Additionally, the inhibition rate (%) was determined using the formula: 100 × [(colony diameter on PDA) − (colony diameter under stress)]/(colony diameter on PDA). Each assay included three technical replicates.

### 2.10. Cell Wall Carbohydrate Composition Determination

Cell wall extraction was performed following a previously described procedure with some modifications [33]. Colonies of WT and HP mutant lines were cultured on PDA plates with cellophane for 2 days to obtain fresh mycelia. About 0.02 g mycelial tissue was harvested and disrupted in 500 μL of 10 mM Tris-Cl (pH8) using three glass beads and the TissueLyser III (QIAGEN, Hilden, Germany) for four cycles of 20 s each, with 20 s cooling intervals on ice. The resulting cell suspension was collected, and the glass beads were thoroughly rinsed with cold Tris buffer three times to recover residual material. The combined supernatant and wash fragments were centrifuged at 3800× *g* for 5 min. The resulting pellet, representing the cell wall fraction, was subsequently washed three additional times with cold deionized water.

The cell wall pellet was suspended in 200 μL of 2N-trifluoroacetic acid (TFA, Sigma-Aldrich, St. Louis, MO, USA) and hydrolyzed in sealed tubes at 121 °C for 1 h. following hydrolysis, TFA was evaporated under nitrogen gas, and the resulting dry samples were resuspended in 1 mL of MilliQ water. The samples were then centrifuged at the maximum speed for 10 min, and the supernatant was collected for quantification of monomeric sugars.

Monosaccharide concentrations were determined using high-performance anion-exchange liquid chromatography (HPAELC) on a Dionex ICS-5000 system equipped with Dionex CarboPac PA1 analytical column (Thermo Fisher Scientific, Waltham, MA, USA) and corresponding guard column. Detection was performed with a pulsed amperometric detector containing a gold working electrode. The mobile phase was delivered at a flow rate of 0.8 mL/min, using Nanopure water as eluent A (0–35 min), followed by 0.2 M NaOH as eluent B for column washing (35–45 min), and re-equilibration with Nanopure water for 15 min. Individual sugars were identified and quantified by comparison with external calibration standards prepared in serial dilutions.

### 2.11. Bleach Immersion and Ultraviolet (UV) Irradiation Assays on Sclerotia

Bleach and UV resistance assays were conducted based on a previously described method with slight modifications [31]. For chemical treatment, sclerotia from two-week-old cultures of the WT and HP mutants were immersed in 15% bleach (Cloromax, The Clorox Company, Okland, CA, USA) for 30, 60, or 90 min. For physical stress, sclerotia were exposed to UV irradiation at an energy dose of 9000 mJ/cm^2^ using a TL-2000 Ultraviolet Translinker (Fisherscientific, Hampton, NH, USA) for 10, 20, or 30 min. For each treatment and strain, 45 sclerotia were collected from multiple independent culture plates to avoid biological variation. After treatment, sclerotia were transferred onto three independent PDA plates and incubated at room temperature for 2 days to assess germination. Germination rate was calculated as the proportion of sclerotia that germinated out of the total number of sclerotia treated. Two independent experiments were conducted with similar results.

### 2.12. Ascospores Quantification and Asci Observation

Each mature apothecium of WT and mutant strains was collected into an individual 1.5 mL Eppendorf tube containing 1 mL of sterile distilled water. Samples were vortexed for 3 min to release ascospores, and the resulting suspensions were used for ascospore quantification. For microscopic observation of asci, small sections of apothecia were sliced and gently squashed on glass slides and examined using a ZEISS AXIO Imager M2 microscope (Jena, Germany) with 200× and 630× magnification. Scale bars are included in each image and described in the corresponding figure legend.

### 2.13. Statistical Analysis

Statistical analyses were performed using GraphPad Prism 10 software (GraphPad Software Inc., San Diego, CA, USA: http://www.graphpad.com/, accessed on 11 July 2023). Differences among means were evaluated by one-way ANOVA with Tukey’s post hoc test. Statistically significant differences were indicated by distinct letters.

## 3. Results

### 3.1. Identification and Classification of Hydrophobin Genes (HPs) in S. sclerotiorum

**Figure 1:**
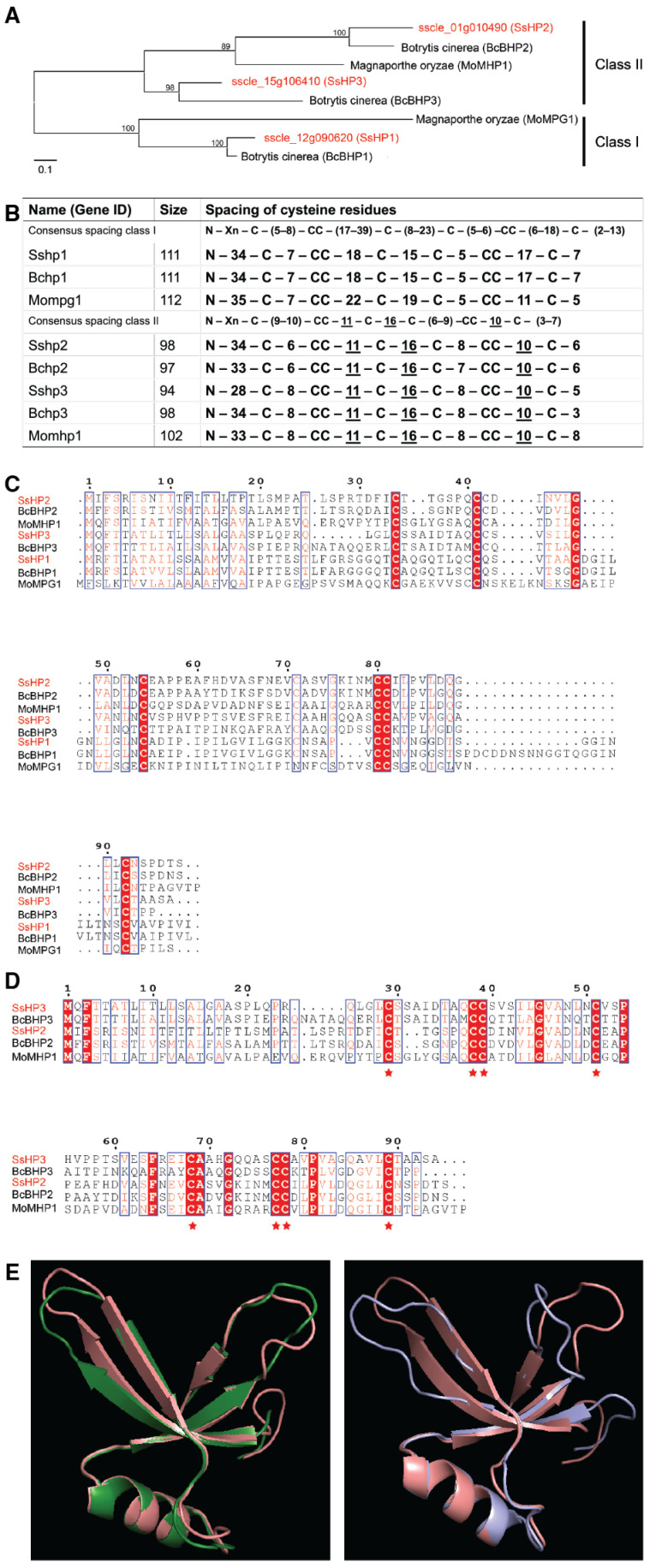
Computational analysis of *S. sclerotiorum* hydrophobins (HPs). (**A**). Phylogenetic analysis of HPs from *S. sclerotiorum* and other filamentous fungi. The tree was constructed using MEGA11, maximum likelihood method and evaluated with 1000 bootstrap replicates. The bootstrap values are labelled above the branches. SsHP1, SsHP2 and SsHP3 are highlighted in red. Homologous HP proteins from representative plant pathogenic fungi, including *M. oryzae* and *B. cinerea*, were identified via NCBI Protein BLAST (2.17.0v) and included in the phylogenetic analysis. The accession numbers of these proteins are APA06279 (SsHP2), XP_001556609 (BcBHP2), XP_003714057 (MoMHP1), APA15871 (SsHP3), XP_001560180 (BcBHP3), XP_003720513 (MoMPG1), APA14292 (SsHP1), and XP_024547321 (BcBHP1). The scale bar is displayed at the bottom. (**B**). HP protein sequence characteristics of representative plant pathogenic fungi. Consensus Cys spacings for class I and class II proteins were adapted from [28]. Protein sizes are indicated. N: N-terminus; Xn: Various number of amino acids; Underlined residues denote conserved spacing. (**C**). Amino acid alignment of SsHP1, SsHP2, SsHP3, and their homologues from representative plant pathogenic fungi. SsHP1, SsHP2 and SsHP3 are highlighted in red. (**D**). Amino acid alignment of class II HPs from *S. sclerotiorum*, *M. oryzae*, and *B. cinerea*. The conserved eight cysteine residues are highlighted in red and marked with asterisks. SsHP2 and SsHP3 are highlighted in red. (**E**). Protein structure alignment via PyMOL 3.1. Left: HP regions of SsHP2 (green) and MoMHP1 (pink), RMSD = 0.311; Right: HP regions of SsHP3 (purple) and MoMHP1 (pink), RMSD = 0.512.

Using BLAST (2.17.0v), three hydrophobin (HP) proteins (*sscle_12g090620*, *sscle_01g010490*, and *sscle_15g106410*) were identified in *S. sclerotiorum*, exhibiting 86.48%, 63.27%, and 51.04%, amino acid similarities with BcBHP1, BcBHP2, and BcBHP3 from *B. cinerea*, respectively (Figure 1A). Accordingly, these genes were named *SsHP1*, *SsHP2*, and *SsHP3*.

*SsHP2* and *SsHP3* share 41.41% and 42.11% similarity, respectively, with the class II HP *MoMHP1* from *M. oryzae*. They share 45.5% identity with each other. Further amino acid alignment of SsHP1, SsHP2, SsHP3 and their homologs in plant pathogenic fungi revealed the presence of the conserved eight cysteine residues (Figure 1B–D). Notably, SsHP2 and SsHP3 contain the conserved inter-Cys spacing characteristic of class II HPs (Figure 1B,D). Their predicted protein structures and hydropathy plots resemble that of MoMHP1, further supporting that they are class II HPs (Figure 1E and Supplementary Figure S1). In contrast, SsHP1 belongs to class I, showing low sequence and structure similarities. SsHP1 is larger in size, consisting of 111 amino acids, and contains considerable variation in the inter-Cys spacing (Figure 1B).

### 3.2. Class I HP Is Required for Normal Sclerotia Development, but Is Not Required for Virulence of S. sclerotiorum

To investigate the biological functions of HPs in *S. sclerotiorum*, a targeted gene knockout (KO) approach (Supplementary Figure S2A) was employed using homologous recombination and protoplast purification to generate pure single-gene deletion mutants in the wild-type (WT; strain 1980) background. Two independent deletion alleles were successfully generated and confirmed by PCR (Supplementary Figure S2B). A 398 bp amplified fragment within the *SsHP1* gene was detected in WT but absent in the KO mutants. Additionally, the presence of the hygromycin-resistance gene (*HYG*) in the KO mutants, but not in WT, further confirmed the successful gene replacement.

**Figure 2:**
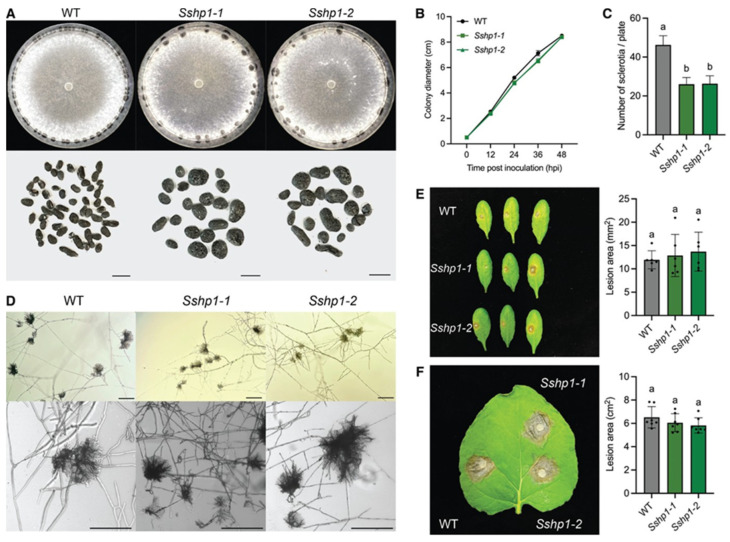
Characterization of the *Sshp1* deletion knockout mutants. (**A**). Colony and sclerotia characteristics of WT and *Sshp1* mutants. All strains were grown on PDA plates, with images captured at 7 days post inoculation (dpi). Sclerotia were harvested from 2-week-old PDA cultures. The scale bar = 0.5 cm. (**B**). Growth of WT and *Sshp1* mutants over 48 h, with colony diameters measured every 12 h. Data represent the means of three replicates, and error bars indicate standard deviations (SDs). (**C**). Number of sclerotia produced by WT and *Sshp1* mutants. Data represent the means of three independent trials. (**D**). Observation of compound appressoria formation in WT and *Sshp1* mutants following transfer from PDA plates to glass slides. The pictures were taken at 2 dpi. The scale bar = 200 μm. (**E**). Disease development of WT and *Sshp1* mutants on unwounded leaves of *A. thaliana*. Representative images at 48 h post inoculation (hpi) (**left**), and corresponding quantification of lesion areas (**right**). (*n* = 6). Each dot represents an individual lesion measured using ImageJ, and different letters indicate statistical significance. Three independent experiments were carried out with similar results. (**F**). Disease development of WT and Sshp1 mutants on unwounded leaves of *N. benthamiana*. Representative images at 36 h post inoculation (hpi) (**left**), and corresponding quantification of lesion areas (**right**). (*n* = 7). Each dot represents an individual lesion measured using ImageJ, and different letters indicate statistical significance. Three independent experiments were carried out with similar results.

Both deletion alleles, *Sshp1-1* and *Sshp1-2*, exhibited similar colony morphology and growth rates as WT, but produced fewer larger sclerotia (Figure 2A–C). Moreover, they formed normal compound appressoria and showed no defects in virulence (Figure 2D–F). These results suggest that SsHP1 is involved in proper sclerotia development but is dispensable for normal growth or pathogenesis in *S. sclerotiorum*.

### 3.3. SsHP2 and SsHP3 Are Essential for Compound Appressoria Function and Full Virulence of S. sclerotiorum

**Figure 3:**
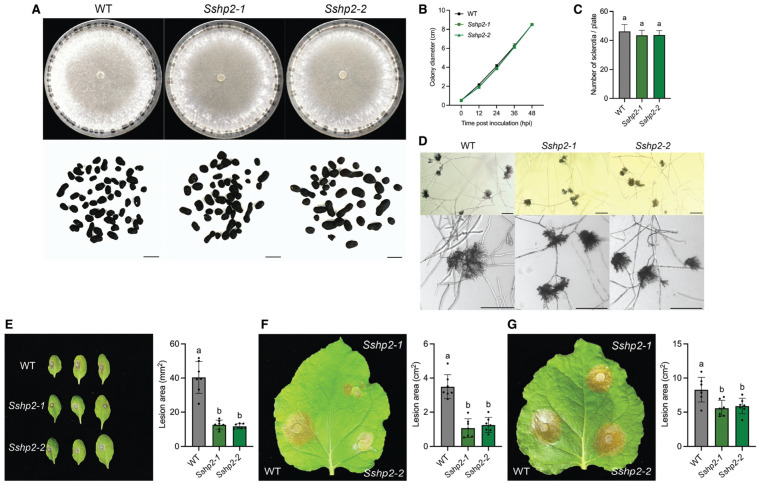
Characterization of the *Sshp2* mutants. (**A**). Colony and sclerotia characteristics of WT and *Sshp2* mutants. All strains were grown on PDA plates, with images captured at 7 dpi. Sclerotia were harvested from 2-week-old PDA cultures. The scale bar = 0.5 cm. (**B**). Growth of WT and *Sshp2* mutants over 48 h, with colony diameters measured every 12 h. Data represent the means of three replicates, and error bars indicate SDs. (**C**). Number of sclerotia produced by WT and *Sshp2* mutants. Data represent the means of three independent trials. (**D**). Observation of compound appressoria formation in WT and *Sshp2* mutants following transfer from PDA plates to glass slides. The pictures were taken at 2 dpi. The scale bar = 200 μm. (**E**). Assessment of infection by WT and *Sshp2* mutants on unwounded leaves of *A. thaliana*. Representative images at 48 hpi (**left**), and corresponding quantification of lesion areas (**right**). (*n* = 7). Each dot represents an individual lesion measured using ImageJ, and different letters indicate statistical significance. Three independent experiments were carried out with similar results. (**F**). Assessment of infection by WT and *Sshp2* mutants on unwounded leaves of *N. benthamiana*. Representative images at 36 hpi (**left**), and corresponding quantification of lesion areas (**right**). (*n* = 7). Each dot represents an individual lesion measured using ImageJ, and different letters indicate statistical significance. Three independent experiments were carried out with similar results. (**G**). Assessment of infection by WT and *Sshp2* mutants on wounded leaves of *N. benthamiana*. Representative images at 36 hpi (**left**), and corresponding quantification of lesion areas (**right**). (*n* = 7). Each dot represents an individual lesion measured using ImageJ, and different letters indicate statistical significance. Three independent experiments were carried out with similar results.

We then investigated the role of class II HPs in *S. sclerotiorum*. Deletion mutants of *SsHP2* and *SsHP3* were generated in the WT background using a similar strategy as with *SsHP1* (Supplementary Figure S2C,D). The two purified *SsHP2* deletion alleles, *Sshp2-1* and *Sshp2-2*, exhibited similar morphological and developmental traits to WT (Figure 3A–D). When inoculated onto host, they showed markedly reduced virulence on both *Arabidopsis thaliana* and *Nicotiana benthamiana* leaves (Figure 3E,F). Notably, the virulence defects were largely rescued when mechanical wounds were introduced on *N. benthamiana* leaves pre-inoculation (Figure 3G). These results indicate that *SsHP2* contributes to appressoria functionality required for successful host penetration, although their appressoria morphology remains normal.

**Figure 4:**
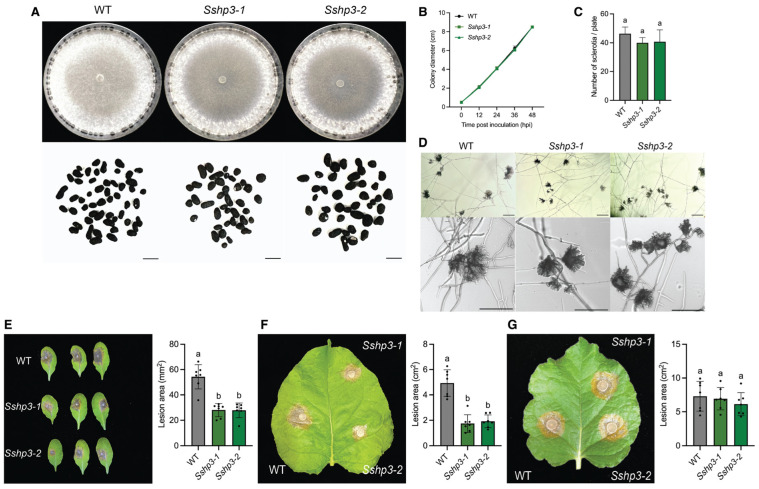
Characterization of the *Sshp3* mutants. (**A**). Colony and sclerotia characteristics of WT and *Sshp3* mutants. All strains were grown on PDA plates, with images captured at 7 dpi. Sclerotia were harvested from 2-week-old PDA cultures. The scale bar = 0.5 cm. (**B**). Growth of WT and *Ssh3* mutants over 48 h, with colony diameters measured every 12 h. Data represent the means of three replicates, and error bars indicate SDs. (**C**). Number of sclerotia produced by WT and *Sshp3* mutants. Data represent the means of three independent trials. (**D**). Observation of compound appressoria formation in WT and *Sshp3* mutants following transfer from PDA plates to glass slides. The pictures were taken at 2 dpi. The scale bar = 200 μm. (**E**). Assessment of infection by WT and *Sshp3* mutants on unwounded leaves of *A. thaliana*. Representative images at 48 hpi (**left**), and corresponding quantification of lesion areas (**right**). (*n* = 7). Each dot represents an individual lesion measured using ImageJ, and different letters indicate statistical significance. Three independent experiments were carried out with similar results. (**F**). Assessment of infection by WT and *Sshp3* mutants on unwounded leaves of *N. benthamiana*. Representative images at 36 hpi (**left**), and corresponding quantification of lesion areas (**right**). (*n* = 7). Each dot represents an individual lesion measured using ImageJ, and different letters indicate statistical significance. Three independent experiments were carried out with similar results. (**G**). Assessment of infection by WT and *Sshp3* mutants on wounded leaves of *N. benthamiana*. Representative images at 36 hpi (**left**), and corresponding quantification of lesion areas (**right**). (*n* = 7). Each dot represents an individual lesion measured using ImageJ, and different letters indicate statistical significance. Three independent experiments were carried out with similar results.

Similarly, deletion mutants of *SsHP3* displayed normal vegetative growth (Figure 4A–D). Their compound appressoria appeared functionally impaired, resulting in reduced lesion sizes on unwounded leaves, whereas lesion sizes on wounded leaves remained comparable to WT across different plant hosts (Figure 4E–G). In summary, these results demonstrate that HPs play diverse roles in the biology of *S. sclerotiorum*. Notably, class II HPs are critical for host penetration and full virulence.

### 3.4. Characterization of the Sshp2 Sshp3 Double Mutants

**Figure 5:**
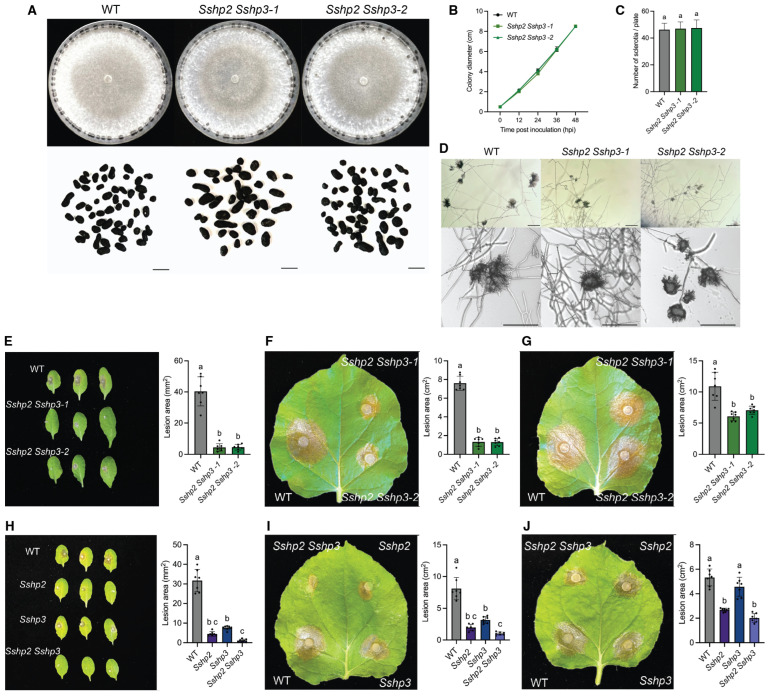
Characterization of the *Sshp2 Sshp3* double mutants. (**A**). Colony and sclerotia characteristics of WT and *Sshp2 Sshp3* double mutants. All strains were grown on PDA plates, with images captured at 7 dpi. Sclerotia were harvested from 2-week-old PDA cultures. The scale bar = 0.5 cm. (**B**). Growth of WT and *Sshp2 Sshp3* double mutants over 48 h, with colony diameters measured every 12 h. Data represent the means of three replicates, and error bars indicate SDs. (**C**). Number of sclerotia produced by WT and *Sshp2 Sshp3* double mutants. Data represent the means of three independent trials. (**D**). Observation of compound appressoria formation in WT and *Sshp2 Sshp3* double mutants following transfer from PDA plates to glass slides. The pictures were taken at 2 dpi. The scale bar = 200 μm. (**E**). Assessment of infection by for WT and *Sshp2 Sshp3* double mutants on unwounded leaves of *A. thaliana*. Representative images at 48 hpi (**left**), and corresponding quantification of lesion areas (**right**). (*n* = 7). Each dot represents an individual lesion measured using ImageJ, and different letters indicate statistical significance. Three independent experiments were carried out with similar results. (**F**). Assessment of infection by WT and *Sshp2 Sshp3* double mutants on unwounded leaves of *N. benthamiana*. Representative images at 36 hpi (**left**), and corresponding quantification of lesion areas (**right**). (*n* = 7). Each dot represents an individual lesion measured using ImageJ, and different letters indicate statistical significance. Three independent experiments were carried out with similar results. (**G**). Assessment of infection by WT and *Sshp2 Sshp3* double mutants on wounded leaves of *N. benthamiana*. Representative images at 36 hpi (**left**) and corresponding quantification of lesion areas (**right**). (*n* = 7). Each dot represents an individual lesion measured using ImageJ, and different letters indicate statistical significance. Three independent experiments were carried out with similar results. (**H**). Assessment of infection by WT and all *HP* mutants on unwounded leaves of *A. thaliana*. Representative images at 48 hpi (**left**) and corresponding quantification of lesion areas (**right**). (*n* = 7). Each dot represents an individual lesion measured using ImageJ, and different letters indicate statistical significance. Three independent experiments were carried out with similar results. (**I**). Assessment of infection by WT and all *HP* mutants on unwounded leaves of *N. benthamiana*. Representative images at 36 hpi (**left**), and corresponding quantification of lesion areas (**right**). (*n* = 7). Each dot represents an individual lesion measured using ImageJ, and different letters indicate statistical significance. Three independent experiments were carried out with similar results. (**J**). Assessment of infection by WT and all HP mutants on wounded leaves of *N. benthamiana*. Representative images at 24 hpi (**left**), and corresponding quantification of lesion areas (**right**). (*n* = 7). Each dot represents an individual lesion measured using ImageJ, and different letters indicate statistical significance. Three independent experiments were carried out with similar results.

Given the essential roles of the two class II HPs in *S. sclerotiorum* virulence, we further generated *Sshp2 Sshp3* double KO mutants to test their redundancy (Supplementary Figure S2E,F). The double KO mutants were morphologically similar to both the WT and single KO mutants, exhibiting normal vegetative growth but producing morphologically abnormal appressoria that were noticeably more compact (Figure 5A–D). Notably, the double mutant exhibited more severe virulence defects than single mutants (Figure 5E,F,H,I). Although mechanical wounding of *N. benthamiana* leaves partially alleviated these defects, the lesions caused by the double mutants remained significantly smaller than those caused by the WT, suggesting their contribution in virulence is beyond host penetration (Figure 5G,J).

**Figure 6:**
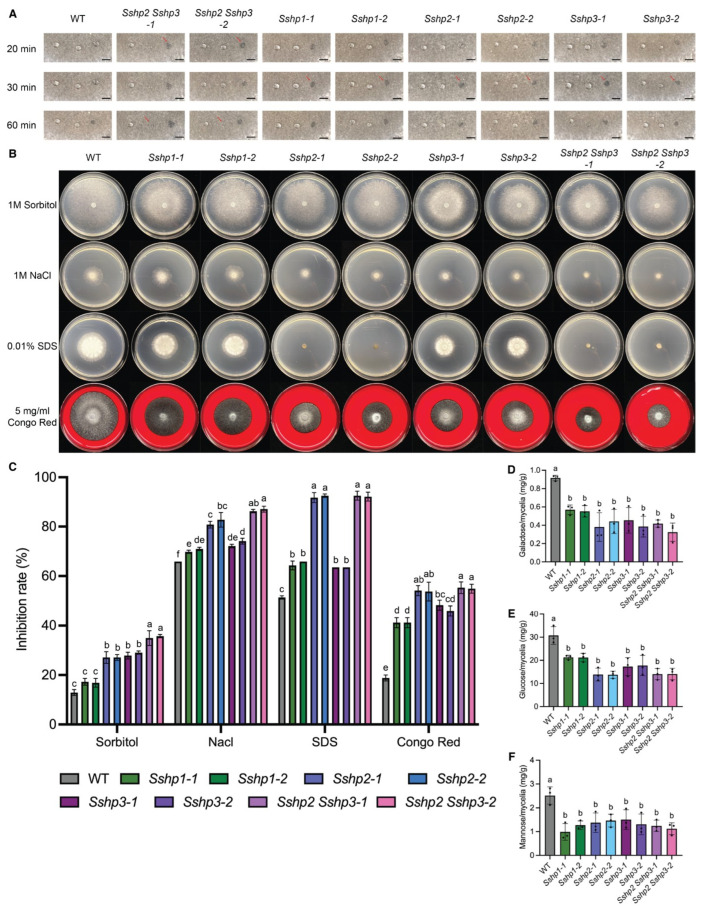
HPs contribute to surface hydrophobicity, cell wall integrity, and stress tolerance of *S. sclerotiorum*. (**A**). Surface hydrophobicity of WT and all HP mutants. 10 μL of sterile distilled water (**left**), 0.2%SDS (**middle**), and 2%SDS (**right**) were placed on the colony surface of WT and mutants. The pictures were taken at indicated time points. Red arrows indicate the soaked phenotype. The scale bar = 0.5 cm. Three independent experiments were carried out with similar results. (**B**). Colony morphology of WT and all HP mutants under different osmotic stressors and cell wall inhibitors. Representative photos were taken at 48 hpi. Three independent experiments were carried out with similar results. (**C**). The inhibition rate of hyphal growth under various stressors and cell wall inhibitors. Inhibition rate = 100% × [(colony diameter on PDA) − (colony diameter under stress)]/(colony diameter on PDA). Error bars show SDs, and letters represent statistical significance. (**D**–**F**). Cell wall composition of WT and mutants. The amount of galactose (**D**), glucose (**E**), and mannose (**F**) per gram of mycelia with three replicates per group. Error bars show SDs, and letters represent statistical significance.

As HPs can self-assemble to form hydrophobic coatings, we tested the hydrophobicity of the *HP* mutants. When 2% SDS was applied to colony surfaces, the liquid droplets penetrated the mycelia of the *Sshp2 Sshp3* double KO mutants within 20 min, while the time for the single *HP* mutants was approximately 30 min. As with the WT control, the droplets persisted on the surface. This demonstrated that the mutants exhibit increased wettability compared with WT. At a lower concentration (0.2% SDS), the droplets soaked into the double KO colonies within 60 min but remained beaded on the WT and single mutants, further confirming the enhanced wettability of the double mutants (Figure 6A).

Since oxalic acid (OA) is a key determinant of *S. sclerotiorum* virulence, we next examined whether HP mutants were impaired in OA production. An OA accumulation assay was performed by inoculating the different strains on PDA supplemented with bromophenol blue. Both WT and HP mutants displayed similar color changes, with plates changing from violet to yellow at 2 days post inoculation (dpi) and reverting to violet by 8 dpi (Supplementary Figure S3A). These results suggest that neither OA production nor its subsequent degradation is affected by loss of HP function.

Taken together, these results suggest that *SsHP2* and *SsHP3* act redundantly in the functionality of compound appressoria and promotion of surface hydrophobicity, both of which are critical for fungal full virulence.

### 3.5. HPs Are Required for Cell Wall Integrity and Stress Resistance in S. sclerotiorum

To further explore the roles of HPs in *S. sclerotiorum*, we assessed the mutants in responses to various stress conditions. WT and *HP* mutant strains were grown on PDA media supplemented with the cell wall inhibitors Congo Red (CR) or SDS, as well as the osmotic stressors NaCl and Sorbitol. Cell wall inhibitors and osmotic stressors restricted the growth of mutant strains to varying degrees (Figure 6B). Compared with WT, all mutants exhibited increased sensitivity to these reagents, especially with *Sshp2* and the double mutants displaying the most pronounced defects (Figure 6B,C). These results indicate that HPs, particularly class II members, are essential for maintaining cell wall integrity and conferring resistance to osmotic stress.

### 3.6. HPs Contribute to Maintaining Proper Cell Wall Composition of S. sclerotiorum

While the role of HPs in mediating cell wall composition has been reported in *S. commune*, it remains uncharacterized in Ascomycetes [21]. In *S. sclerotiorum*, the HP mutants exhibited hypersensitivity or formed smaller colonies on SDS and CR plates, suggesting defects in cell wall biogenesis (Figure 6B). We therefore analyzed the cell wall composition of these mutants. As shown in Figure 6D–F, the amounts of galactose, glucose, and mannose released from the cell walls of both single and double mutants were significantly lower than those from WT cell walls. Notably, compared with class II HPs, disruption of *SsHP1* resulted in a pronounced reduction in mannose but moderate effects on glucose and galactose. These findings suggest that *S. sclerotiorum* class II HPs broadly influence overall cell wall carbohydrate content and the crosslinking between wall layers, whereas class I HP predominantly affects mannose, the major sugar in the outer layer.

### 3.7. HPs Are Essential for Sclerotia Stress Tolerance

**Figure 7:**
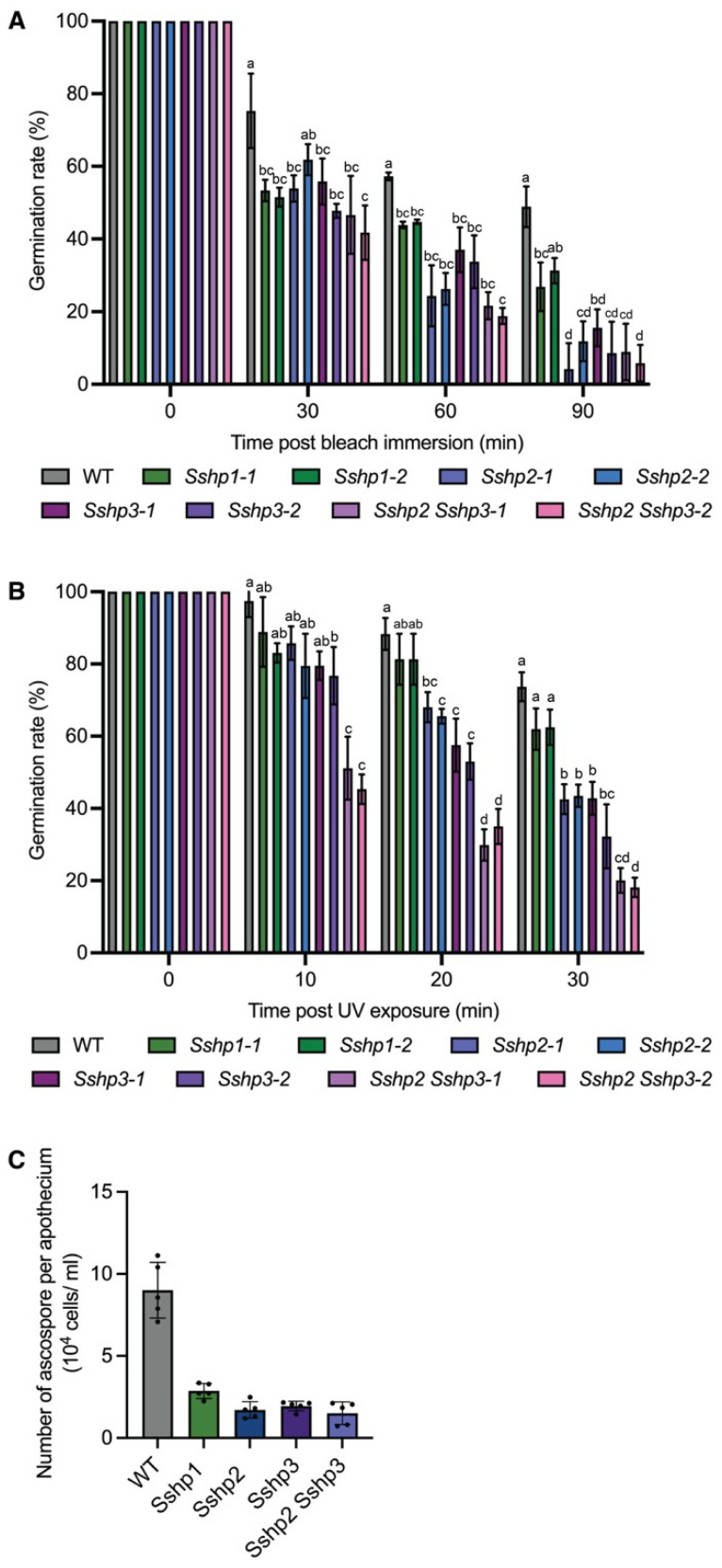
HPs are involved in *S. sclerotiorum* sclerotia survival and ascospore dispersal. (**A**). The sensitivity of WT and mutants to immersion in 15% bleach over time. Germination rate was determined by dividing the number of germinated sclerotia by the total number of treated sclerotia. Error bars show SDs, and letters represent statistical significance. (**B**). The sensitivity of WT and mutants to UV irradiation over time. Germination rate was determined by dividing the number of germinated sclerotia by the total number of treated sclerotia. Error bars show SDs, and letters represent statistical significance. (**C**). Quantification of ascospores released from each apothecium. The dots represent the number of ascospores measured using the hemocytometer. Error bars show SDs (*n* = 5), and letters represent statistical significance.

Since sclerotia are hyphal aggregates that protect *S. sclerotiorum* against physical, chemical, and biological stresses [1], we tested whether sclerotia from HP-deficient mutants could withstand toxic bleach treatment or UV irradiation and germinate properly. Compared with the WT, myceliogenic germination rates were significantly reduced in the mutants after varying durations of bleach immersion and UV exposure (Figure 7A,B). Notably, following 60 min of bleach treatment or 20 min of UV irradiation, *Sshp2 Sshp3* sclerotia exhibited approximately 35% and 37% reductions in germination rate, respectively. Taken together, these results indicate that HPs are essential for sclerotia survival under both physical and chemical stresses. HPs play critical roles to support the long-term survival of the sclerotia in nature.

### 3.8. HPs Involved in Ascospore Dispersal of S. sclerotiorum

In *B. cinerea*, HPs have been reported to play important roles in apothecium development [13]. To assess whether they serve similar functions in *S. sclerotiorum*, we examined apothecia formation in the HP mutants. All the HP mutants produced morphologically normal apothecia. However, each apothecium released significantly fewer ascospores compared with the WT (Figure 7C). Microscopic examination of asci morphology revealed no detectable defects in asci development across the mutants (Supplementary Figure S3B). Taken together, these results indicate that while HPs are dispensable in apothecium and ascus morphogenesis, they are critical for the biophysical process of ascospore dispersal.

## 4. Discussion

HPs are essential in the life cycle of filamentous fungi. They cover the aerial hyphae and spores to enable hydrophobicity, allowing them to grow into air or disperse into new environment during differentiation or dissemination [34]. HPs also play important roles in host–pathogen interactions. Although HPs have been extensively studied in many fungal species, their roles in *S. sclerotiorum* remain poorly understood. In this study, we identified and functionally characterized three *HP* genes in *S. sclerotiorum*: the sole class I HP, *SsHP1*, and two class II HPs, *SsHP2* and *SsHP3*. Several features support their classification as members of the HP family: they encode small proteins of approximately 100 amino acids, contain an N-terminal signal peptide indicative of secretion, and display the conserved spacing of eight cysteine residues. In addition to sequence similarity with HPs from other fungi, the *S. sclerotiorum* HPs share known functional properties, including conferring surface hydrophobicity and contributing to protection against environmental stress.

Using homologous recombination, we generated single deletion mutants of all *HP* genes in *S. sclerotiorum* and analyzed their roles in growth, development, and plant infection. SsHP1 was found to primarily regulate sclerotia development, while being dispensable for vegetative growth and virulence. In contrast, SsHP2 and SsHP3 play broader roles in fungal biology, being essential for proper compound appressoria function and full virulence. In addition, by employing a mycelial fusion strategy, double mutants lacking both class II HP genes were successfully generated, enabling an assessment of functional redundancy that might be masked in single mutants. The *Sshp2 Sshp3* double mutants exhibited more severe phenotypes, including defective appressoria formation, markedly decreased virulence, compromised cell wall integrity, and reduced sclerotia stress tolerance. These results indicate the functional redundancy and overlapping contributions of SsHP2 and SsHP3 to host penetration, colonization, and fungal fitness, highlighting their multifaceted roles in *S. sclerotiorum* biology.

Our study in *S. sclerotiorum* revealed that both class I and class II HPs contribute to surface hydrophobicity, while also performing distinct functions: SsHP1 primarily regulates sclerotia development, whereas SsHP2 and SsHP3 are more critical to appressoria formation and virulence. These findings are consistent with previous observations in *M. oryzae* but also diverge in specific aspects. In *M. oryzae*, both class I and II HPs are required for surface hydrophobicity, appressoria development and virulence; the class I HP contributes to rodlet layer formation and surface sensing, whereas the class II HP plays a more central role in plant colonization [25,26]. Taken together, these results point to both conserved and divergent roles of HPs across fungal species.

The orthologs of SsHP1, SsHP2, and SsHP3 have also been characterized in the closely related pathogenic species *B. cinerea*. However, our results reveal striking differences in HP function between these two closely related fungi. In *B. cinerea*, HPs are largely dispensable for surface hydrophobicity and virulence but play critical roles in sexual development, particularly apothecia formation [13,30]. In contrast, all *S. sclerotiorum* HP mutants formed normal apothecia and asci, but released significantly fewer ascospores than the WT. This indicates that HPs are not required for the morphogenesis of sexual structures in *S. sclerotiorum* but are crucial for the biophysical process of ascospore dispersal. Given their ability to modify surface hydrophobicity and influence biophysical properties, HPs may facilitate ascus dehiscence or reduce adhesion forces that otherwise restrict spore discharge. They may also contribute to the mechanical tension of ascus or spore cell walls, promoting efficient spore dispersal. This reveals a previously unrecognized role for HPs in fungal spore dissemination. The contrasting outcomes in *S. sclerotiorum* and *B. cinerea* likely reflect differences in both ecological specialization and reproductive strategies. *S. sclerotiorum* relies on long-living sclerotia for survival and infect hosts through compound appressoria, while *B. cinerea* depends heavily on massive number of conidia for dispersal and reproduction. It is also possible that the *HP* expression pattern differ between the two species, with *B. cinerea HPs* being predominantly expressed during sexual development, while *S. sclerotiorum HPs* might be more tightly associated with infection-related processes [13]. This divergence highlights the evolutionary flexibility of HP function. Even within closely related fungi, HPs have been co-opted to fulfill distinct roles in development, reproduction, and virulence.

In this study, we also revealed novel roles of *S. sclerotiorum* HPs in maintaining cell wall integrity and composition, with the double HP mutants exhibiting the most severe cell wall-related defects. To our knowledge, this represents the first demonstration of HPs influencing cell wall organization in a plant pathogenic fungus. A robust cell wall is essential not only for appressoria-mediated host penetration but also for resisting against host-derived stresses. Comparable observations have been reported in the Basidiomycete *S. commune*, where deletion of the major HP *SC3* led to pronounced alterations in cell wall composition, including increased secretion of soluble β-glucan mucilage and a reduction in chitin-linked glucans [21]. Moreover, in the insect pathogenic fungus *Beauveria bassiana*, loss of *HP* genes down-regulated cell wall integrity pathway genes and impaired plant roots colonization by the insect [35]. In *S. sclerotiorum*, SsHP1 appears to function as a “surface organizer”, primarily affecting mannose and consequently influencing the assembly of mannoproteins in the outer cell wall layer. In contrast, Class II HPs act as “global cell wall stabilizer”, as class II HP mutants exhibited marked reductions in all major sugars, including glucose, galactose and mannose. These alterations in cell wall composition align with observed phenotypes: class I HP mainly support survival-related traits with limited role in infection, whereas class II HPs serve major roles in maintaining cell wall integrity and regulating infection-related development. The mechanisms by which HPs affect cell wall integrity remain to be elucidated. One possibility is direct structural interaction with cell wall components. Given their amphipathic properties, HPs may cross-link with glucans or mannans, influencing cell wall stability and organization. Alternatively, HPs may act indirectly through signaling pathways, potentially interacting with the cell wall integrity (CWI) MAPK cascade, which monitors and responses to cell wall stress.

In summary, our findings broaden the understanding of HPs by linking them to sclerotia development, cell wall integrity, stress tolerance and virulence in *S. sclerotiorum*. The discovery that HPs contribute to cell wall integrity, virulence and ascospore dispersal in *S. sclerotiorum* has important implications for understand host–pathogen interactions. HPs may act directly by interacting with cell wall polymers or indirectly by modulating cell wall integrity signaling pathways. Moreover, their unique biophysical properties that alter surface hydrophobicity likely facilitate ascospore dispersal. However, the precise mechanisms underlying these effects remain unclear and require further investigation. In addition, comparative analyses of *HP* gene expression patterns in *S. sclerotiorum* and other species could help elucidate potential functional diversification among HPs across species. Given their multifunctional roles in both development and virulence, HPs could be promising targets for novel disease control strategies. Approaches such as host-induced gene silencing could provide innovative avenues for sustainable *S. sclerotiorum* disease management. Nevertheless, several practical limitations should be considered. For example, functional redundancy among HP members may reduce the effectiveness of single-gene targeting, and the intrinsic stability of HPs allows them to form persistent protein layers that are difficult to disrupt. Therefore, effective control may require multiple targeting or integrated management approaches to overcome these challenges and achieve durable disease suppression.

## Data Availability

The original contributions presented in this study are included in the article/Supplementary Materials. Further inquiries can be directed to the corresponding author.

## Supplementary Materials

The following supporting information can be downloaded at: https://www.mdpi.com/article/10.3390/pathogens14111131/s1, Figure S1: SsHP1 is class I HP, while SsHP2 and SsHP3 belong to class II; Figure S2: Two independent deletion alleles of *Sshp1*, *Sshp2*, and *Sshp3* were obtained through homologous recombination; Figure S3: Oxalic acid accumulation, apothecia formation, and asci development in HP mutants. Table S1: List of primers used in this study.
