# Supplementary material for: Reverse Genetic Analyses of Hydrophobins in Sclerotinia sclerotiorum Revealed Their Diverse Roles in Development, Environmental Survival, and Virulence
Source: Pathogens. 2025 Nov 6;14(11):1131. doi: 10.3390/pathogens14111131 (PMC12655489; doi:10.3390/pathogens14111131)
Supplement: Supplementary file 1 [file pathogens-14-01131-s001.zip › pathogens-3956112-supplementary.pdf]

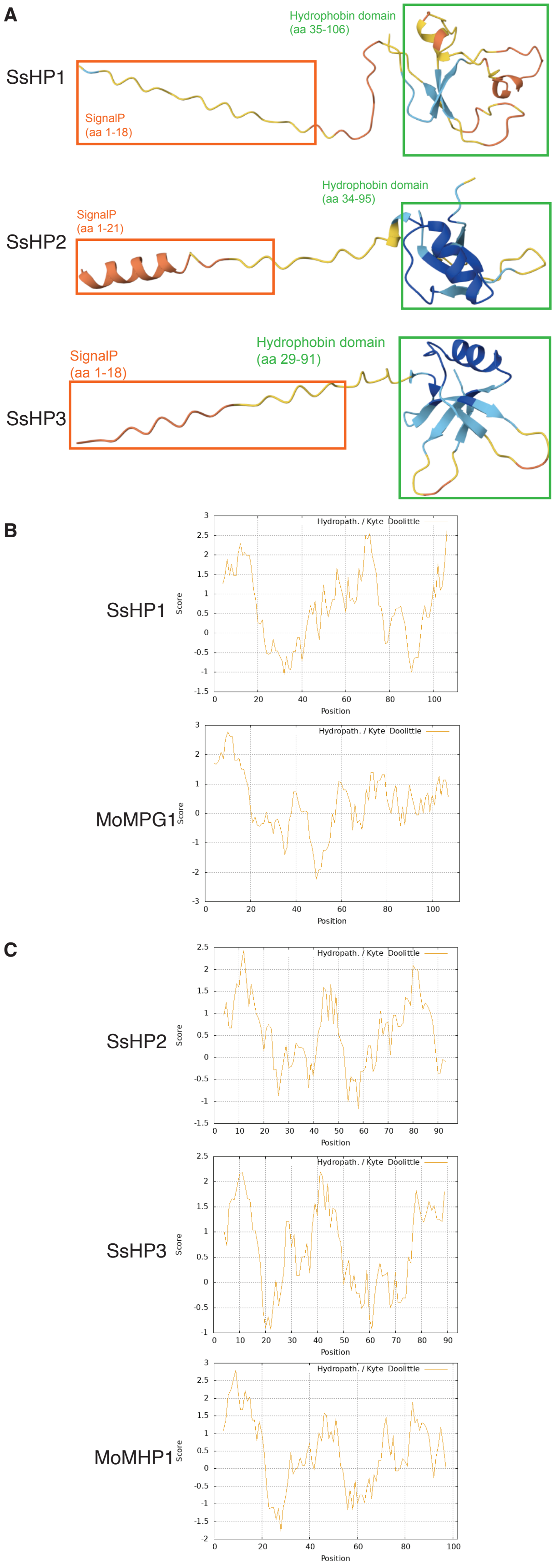

Supplemental Figure S1. SsHP1 is class I HP, while SsHP2 and SsHP3 belong to class II.
A. Protein structures of SsHP1, SsHP2, and SSHP3 as predicted by AlphaFold3. SignalP region and HP domain are highlighted in orange and green boxes respectively with amino acid (aa) positions labeled below.
B. Hydropathy plot of SsHP1, generated using ExpPASy ProtScale (https://web.expasy.org/protscale/) based on the Kyte and Doolittle algorithm, was compared with class I HP MoMPG1 from *M. oryzae*. Hydrophobic regions are displayed above the x-axis, while hydrophilic regions appear below.
C. Hydropathy plot of SsHP2 and SsHP3, generated using ExpPASy ProtScale based on the Kyte and Doolittle algorithm, were compared with class II HP MoMHP1 from *M. oryzae*. Hydrophobic regions are displayed above the x-axis, while hydrophilic regions appear below.

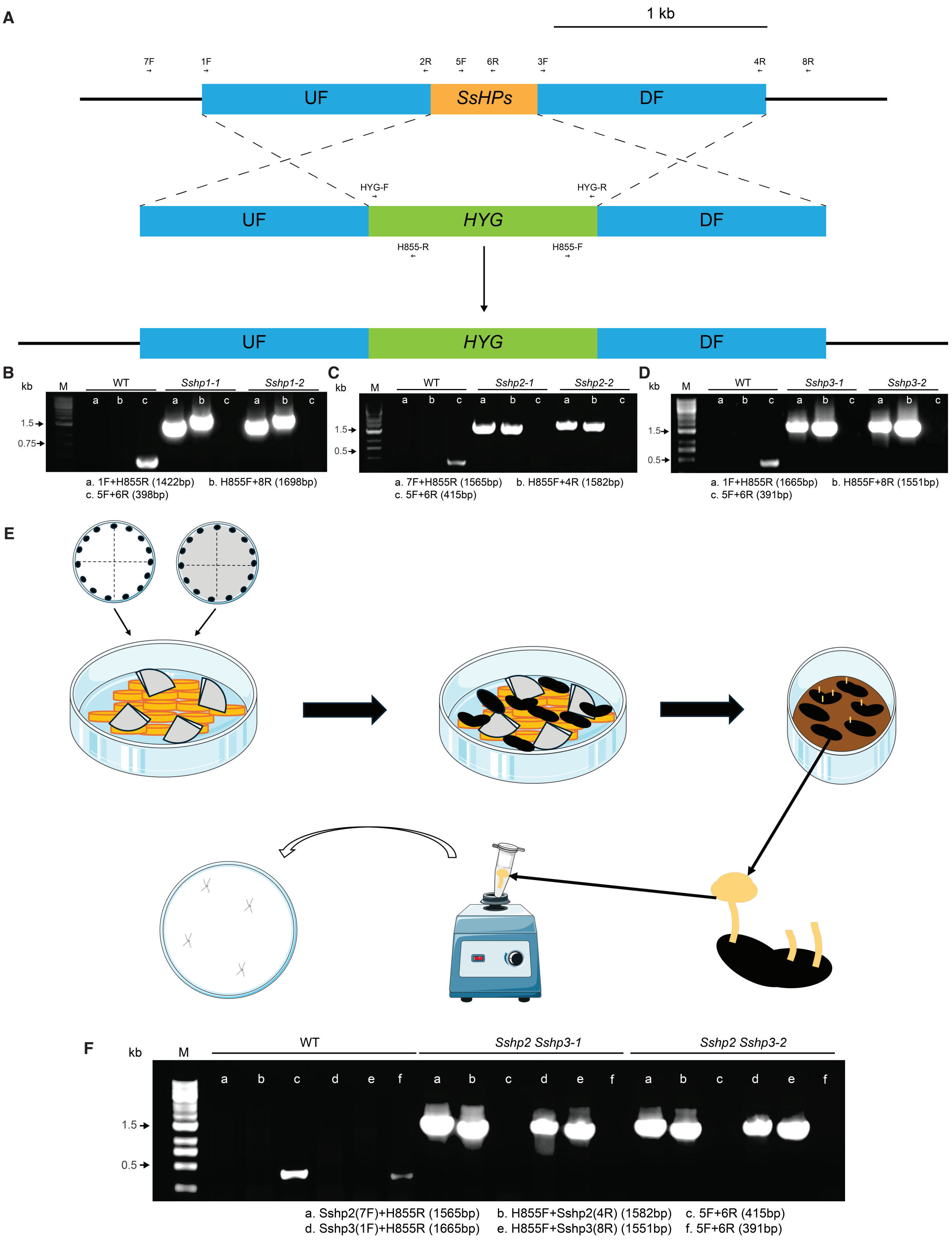
A
1 kb
7F
1F
2R
5F
6R
3F
4R
8R
UF
SsHPs
DF
HYG-F
HYG-R
UF
HYG
DF
H855-R
H855-F
UF
HYG
DF
B
kb
M
WT
Sshp1-1
Sshp1-2
a b c a b c a b c
1.5
0.75
a. 1F+H855R (1422bp) b. H855F+8R (1698bp)
c. 5F+6R (398bp)
C
kb
M
WT
Sshp2-1
Sshp2-2
a b c a b c a b c
1.5
0.5
a. 7F+H855R (1565bp) b. H855F+4R (1582bp)
c. 5F+6R (415bp)
D
kb
M
WT
Sshp3-1
Sshp3-2
a b c a b c a b c
1.5
0.5
a. 1F+H855R (1665bp) b. H855F+8R (1551bp)
c. 5F+6R (391bp)
E
F
kb
M
WT
Sshp2 Sshp3-1
Sshp2 Sshp3-2
a b c d e f a b c d e f a b c d e f
1.5
0.5
a. Sshp2(7F)+H855R (1565bp) b. H855F+Sshp2(4R) (1582bp) c. 5F+6R (415bp)
d. Sshp3(1F)+H855R (1665bp) e. H855F+Sshp3(8R) (1551bp) f. 5F+6R (391bp)

Supplemental Figure S2. Two independent deletion alleles of *Sshp1*, *Sshp2*, and *Sshp3* were obtained through homologous recombination.

A. The SsHP genes and HYG gene are presented as orange and green rectangles, respectively. The primers labelled in the diagram were used for mutant screening. The scale is shown at the bottom.

B. PCR verification of *sscle_12g090620* (*SsHP1*) gene disruption. Genomic DNA isolated from WT and independent deletion mutant alleles *Sshp1-1* and *Sshp1-2* were used as PCR templates. Three pairs of primers were designed to verify the upstream and downstream of the hygromycin resistance gene (1F + H855R and H855F + 8R) and SsHP1 deletion (5F + 6R). The amplicon sizes are indicated in brackets. M shows the lane for DNA marker.

C. PCR verification of *sscle_01g010490* (*SsHP2*) gene disruption. Genomic DNA isolated from WT and independent deletion mutant alleles *Sshp2-1* and *Sshp2-2* were used as PCR templates. Three pairs of primers were designed to verify the upstream and downstream of the hygromycin resistance gene (7F + H855R and H855F + 4R) and SsHP2 deletion (5F + 6R). The amplicon sizes are indicated in brackets. M shows the lane for DNA marker.

D. PCR verification of *sscle_15g106410* (*SsHP3*) gene disruption. Genomic DNA isolated from WT and independent deletion mutant alleles *Sshp3-1* and *Sshp3-2* were used as PCR templates. Three pairs of primers were designed to verify the upstream and downstream of the hygromycin resistance gene (1F + H855R and H855F + 8R) and SsHP2 deletion (5F + 6R). The amplicon sizes are indicated in brackets. M shows the lane for DNA marker.

E. Flow chart of *Sshp2 Sshp3* double KO mutant generation through mycelial fusion. *Sshp2* and *Sshp3* single KO strains were placed face-to-face in direct contact and co-inoculated onto carrot medium. The large sclerotia produced on the carrot medium were collected, washed, sterilized, and then induced to generate apothecia. Ascospores from individual apothecium were vortexed for collection and plated onto PDA plates with hygromycin B. The genotype of single colonies was confirmed by PCR. Images adapted from Servier Medical Art (https://smart.servier.com/), licensed under CC BY 4.0 (https://creativecommons.org/licenses/by/4.0/).

F. PCR verification of *SsHP2* and *SsHP3* genes disruption. Genomic DNA isolated from WT and deletion mutant alleles *Sshp2 Sshp3-1* and *Sshp2 Sshp3-2* were used as PCR templates. Six pairs of primers were used to verify the upstream and downstream of the hygromycin resistance gene and targeted genes deletion. The amplicon sizes are indicated in brackets. M shows the lane for DNA marker.

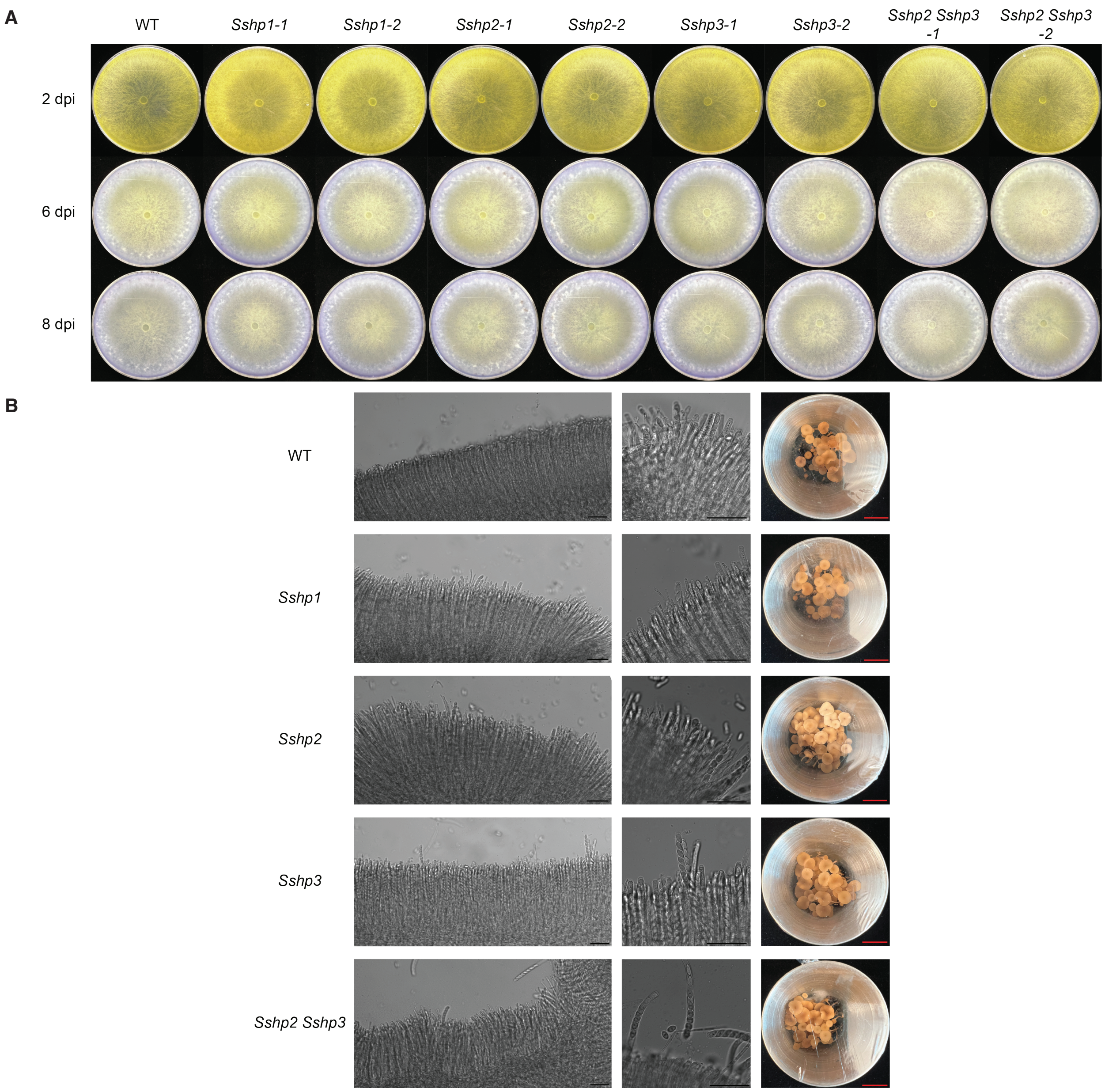

Supplemental Figure S3. Oxalic acid accumulation, apothecia formation, and asci development in HP mutants
A. WT and HP mutants were inoculated on PDA medium supplemented with 50 mg/L bromophenol blue. Acidification, indicating oxalic acid production, is shown by the color change from violet to yellow. Photos were taken at 2, 6 and 8 dpi, respectively.
B. Apothecia induction and asci development in WT and HP mutants. Black scale bar = 50 μm; red scale bar = 1 cm.

Supplemental Table S1. List of primers used in this study.

Primer name	DNA sequence (5' to 3')	Purpose
SsHP1-1F	GTTTTCTCGCTACCACCAAG	*SsHP1* knockout
SsHP1-2R	cgactctagaggatcccggGTGTTGTGTTTGAAGCGAAG	
SsHP1-3F	gttggtgtcgatgtcagctcGGTACACAGGGAGGAATCAA	
SsHP1-4R	TCCCAGCACCGAGTAATAAT	
SsHP1-5F	ACCACCCTCCTTACAATCAA	
SsHP1-6R	TAGGGAAGACTTACGCTGGT	
SsHP1-7F	GAAAATGGACTTGGCTGCAGC	
SsHP1-8R	CTACTCAATCCCTCTCTAAGC	
Hygro-F	CCGGGATCCTCTAGAGTCG	
Hygro-R	GAGCTGACATCGACACCAAC	
SsHP2-1F	CTAGTCTCTTTCGCGTTCG	*SsHP2* and *SsHP3* knockout
SsHP2-2R	cgggtaccgagctctttgaaggttgtgGGGATTGAGTGATGGTGTAG	
SsHP2-3F	gccgaccgggatccacttaacgttacCTTGTGCAAATCCGCGTCAG	
SsHP2-4R	GGTGGAGTTGAACGATCAAG	
SsHP2-5F	CACACAACAGACTCCCATC	
SsHP2-6R	CTGGAGAATTGCAGAGGAGAC	
SsHP2-7F	CTGTCTAAAGAGCTAGCTAC	
SsHP2-8R	GATGGTATGTAATCATGCGC	
HYG-F	CACAACCTTCAAAGAGCTCGGTACCCG	
HYG-R	GTAACGTTAAGTGGATCCCGGTCGGC	
SsHP3-1F	GAGGCTGAAGAACAGTAACC	
SsHP3-2R	cgggtaccgagctctttgaaggttgtgGTTGGTGGGTTGGAAGTGATG	
SsHP3-3F	gccgaccgggatccacttaacgttacGAATGGAATTAGTGCGGC	
SsHP3-4R	GTCGAAGCGCACTAATTATC	
SsHP3-5F	GCAATTCACAACCGCAACAC	
SsHP3-6R	CCATTCTAATCTCAGGCTTC	
SsHP3-7F	CATATGTGCGAAGAAGAGG	
SsHP3-8R	GCGAATACTTCACAACCTATC	
H855F	GTCGATGCGACGCAATCGT	Check *HYG gene insertion*
H855R	GAACCATCTTGTCAAACGAC	
